# Antimicrobial, Antioxidant and Anti-Inflammatory Activities of the Mucus of the Tropical Sea Slug *Elysia crispata*

**DOI:** 10.3390/molecules29194593

**Published:** 2024-09-27

**Authors:** Diana Lopes, Eva Cunha, Tiago Conde, Anthony Moreira, Sónia Cruz, Pedro Domingues, Manuela Oliveira, Paulo Cartaxana

**Affiliations:** 1 Laboratory for Innovation and Sustainability of Marine Biological Resources (ECOMARE), Centre for Environmental and Marine Studies (CESAM), Department of Chemistry, University of Aveiro, 3810-193 Aveiro, Portugal; lopes.diana@ua.pt; 2 Centre for Interdisciplinary Research in Animal Health (CIISA), Faculty of Veterinary Medicine, University of Lisbon, 1300-477 Lisboa, Portugal; evacunha@fmv.ulisboa.pt (E.C.); moliveira@fmv.ulisboa.pt (M.O.); 3 Associate Laboratory for Animal and Veterinary Sciences (AL4AnimalS), 1300-477 Lisboa, Portugal; 4 Mass Spectrometry Centre, Associated Laboratory for Green Chemistry of the Network of Chemistry and Technology (LAQV-REQUIMTE), Department of Chemistry, University of Aveiro, 3810-193 Aveiro, Portugal; tiagoalexandreconde@ua.pt (T.C.); p.domingues@ua.pt (P.D.); 5 ECOMARE, CESAM, Department of Biology, University of Aveiro, 3810-193 Aveiro, Portugal; anthony.moreira@ua.pt (A.M.); sonia.cruz@ua.pt (S.C.); 6 Centre for Ecology, Evolution and Environmental Changes (cE3c) & Global Change and Sustainability Institute (CHANGE), Faculty of Sciences, University of Lisbon, Campo Grande, 1749-016 Lisboa, Portugal

**Keywords:** antibiotics, bioactivity, kleptoplasty, marine natural products, *Pseudomonas aeruginosa*, Sacoglossa

## Abstract

*Elysia crispata* (Sacoglossa, Gastropoda) is a tropical sea slug known for its ability to incorporate functional chloroplasts from a variety of green macroalgae, a phenomenon termed kleptoplasty. This sea slug, amenable to laboratory cultivation, produces mucus, a viscous secretion that serves diverse purposes including protection, locomotion, and reproduction. In this study, we profiled the antimicrobial, antioxidant, and anti-inflammatory activities of the mucus of this sea slug. Results revealed inhibitory activity against several bacterial strains, more pronounced for Gram–negative bacteria. Particularly interesting was the strong inhibitory effect against *Pseudomonas aeruginosa*, a bacterial species classified by the WHO as a high-priority pathogen and associated with high-risk infections due to its frequent multidrug-resistant profile. Similar inhibitory effects were observed for the mucus native protein extracts, indicating that proteins present in the mucus contributed significantly to the antimicrobial activity. The mucus also showed both antioxidant and anti-inflammatory activities. The latter activities were associated with the low molecular weight (<10 kDa) fraction of the mucus rather than the native protein extracts. This study opens the way to further research on the biotechnological applications of the mucus secreted by this unique marine organism, particularly as an antimicrobial agent.

## 1. Introduction

The exploration of the marine environment for novel bioactive compounds has emerged as a vibrant and promising field within biotechnology [1,2]. The ocean, with its vast and largely unexplored biodiversity, offers an unparalleled reservoir of unique organisms and metabolites [3]. The marine ecosystem is home to millions of species, many of which have unique biochemical pathways developed through millions of years of evolution, leading to the production of compounds with extraordinary structural diversity and bioactivities [4]. Marine organisms have adapted to various extreme environments, resulting in novel compounds that are not typically found in terrestrial organisms [5,6].

Mucus is a viscous secretion produced by a wide range of marine organisms such as fish, mollusks, and cnidarians. Mucus is a key factor for the adaptation of these organisms, serving several biological functions, such as locomotion, defense, feeding, and reproduction [7,8,9,10]. Within these mucus-secreting marine animals, a clade of sea slugs—the Sacoglossa—commonly referred to as “solar-powered sea slugs” stands out. Some of these sacoglossans are capable of sequestering functional chloroplasts from their prey algae, a mechanism termed kleptoplasty [11]. In the animal kingdom, the capacity for long-term (several weeks to months) maintenance of these photosynthetically competent chloroplasts is a unique characteristic of Sacoglossa [12,13]. The “stolen” chloroplasts—kleptoplasts—confer biological benefits for the host sea slug, including longer survival in periods of food shortage, higher reproductive output, and increased mucus production [14,15,16,17].

*Elysia crispata* (Mörch, 1863) is one of the largest species (up to 15 cm in length) of tropical Sacoglossa, capable of feeding on different macroalgae from which it sequesters long-term functional chloroplasts [18,19]. We observed that sea slugs that were kept under regular light conditions produced higher quantities of mucus when compared to conspecifics reared under very low irradiances that were limited in their photosynthetic capacity [20]. The total carbohydrate concentrations and the viscosity of *E. crispata* mucus were also higher when animals were reared under regular irradiances, indicating that photosynthesis could support the synthesis of metabolites present in the mucus [20].

A recent detailed proteomic analysis of the mucus of the sea slug *E. crispata* identified a high number of proteins with potential enzymatic activity, suggesting possible biotechnological applications [21]. Hydrolases accounted for the majority of proteins with putative enzymatic activity, indicating the need to assess the antimicrobial activity of *E. crispata* mucus [21]. In this study, we screened the antimicrobial activity of the mucus of this sea slug against a variety of Gram–negative and Gram–positive bacteria, including species classified by the World Health Organization (WHO) as high-priority pathogens [22]. Furthermore, we assessed the antioxidant and anti-inflammatory activities of *E. crispata* mucus to determine other potential biotechnological applications.

## 2. Results

### 2.1. Antimicrobial Activity

The antimicrobial potential of mucus samples from the sea slug *E. crispata* was evaluated using 10 μL of mucus against 17 bacterial isolates (Table 1). Mucus samples showed inhibitory activity against most of the strains tested, except for *Staphylococcus pseudintermedius* 93/23 and *Staphylococcus aureus* ATCC 29213, for which no activity was observed. Overall, higher inhibitory activity was observed for Gram–negative bacteria. The highest inhibitory activities observed were against the three strains of *Pseudomonas aeruginosa.* A medium inhibitory activity was observed for two strains of *Listeria monocytogenes* and for *Aeromonas hydrophila* ATCC 7966 (Table 1).

From these 17 bacterial isolates, seven strains were chosen to test the antimicrobial activities of protein extracts (10 μL) in native conditions (Table 2). Similar inhibitory effects were observed with these native extracts compared with those of mucus samples. However, while mucus samples showed no inhibitory effect against *S. aureus* ATCC 29213, a low inhibitory effect was observed for protein extracts. Most relevant was the high inhibitory effect of native protein extracts against the three strains of *P. aeruginosa*, as observed for the mucus samples.

### 2.2. Antioxidant Activity

**Figure 1:**
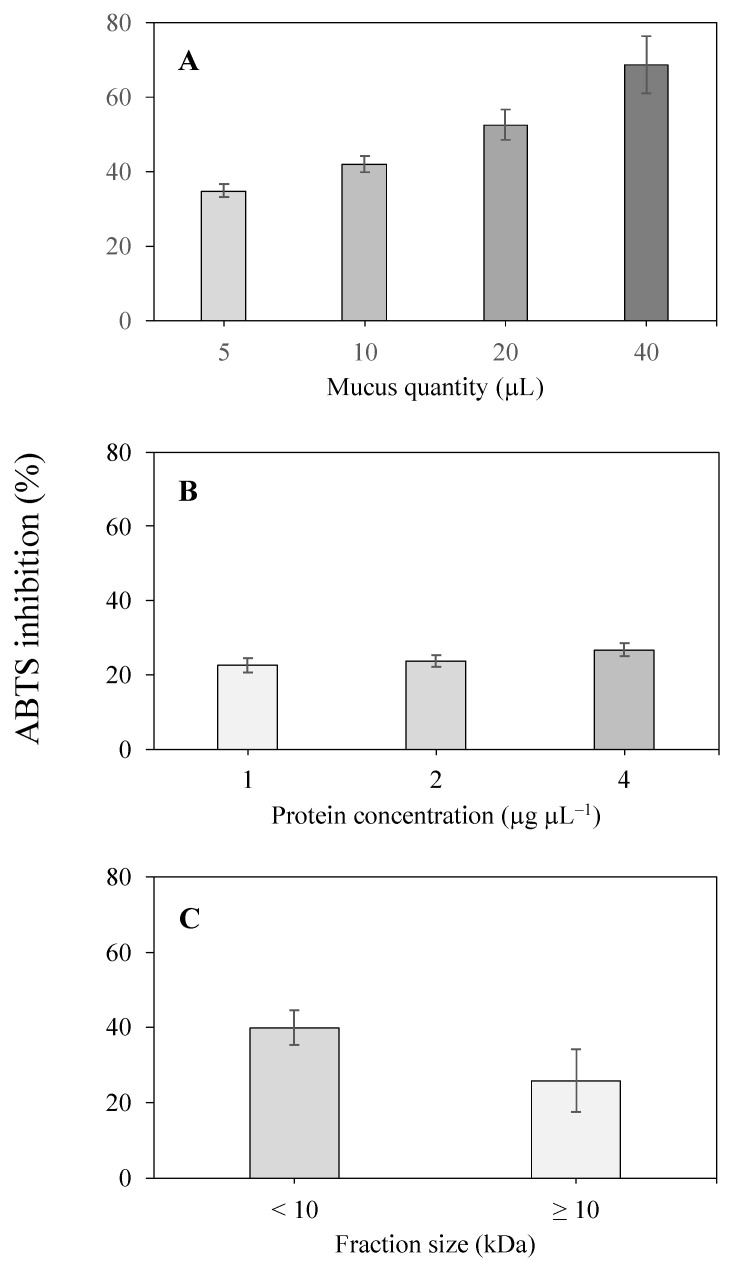
Assessment of the antioxidant activity of mucus samples from the tropical sea slug *Elysia crispata*. (**A**) Antioxidant activity of different volumes of concentrated mucus; (**B**) Antioxidant activity for different protein concentrations of native protein extracts obtained from the sea slug mucus; and (**C**) Antioxidant activity of 10 μL of <10 kDa and ≥10 kDa mucus fractions. The values are displayed as the mean ± standard deviation (*n* = 3).

The antioxidant activity of the mucus of the sea slug *Elysia crispata* was assessed using the ABTS scavenging assay and presented as the percentage of inhibition of the ABTS radical (Figure 1). The volume of mucus used in the assays significantly affected (*p* < 0.001) the observed antioxidant activity (Figure 1A). ABTS radical inhibition of 40 μL of mucus (68.9 ± 7.7%) was significantly higher than of all other tested volumes (in all cases *p* < 0.05). Significantly lower antioxidant activity (34.9 ± 1.8%) was observed for 5 μL of mucus than for 20 and 40 μL (in both cases *p* < 0.01). Intermediate activities were observed for mucus volumes of 10 and 20 μL (42.1 ± 2.3% and 52.6 ± 4.1%, respectively). On the other hand, increased protein concentrations had no significant effect on the antioxidant capacity of the tested native protein extracts, with consistently low inhibitory values over the entire range (22.5 to 26.8%; Figure 1B). Subsequently, the antioxidant capacity was tested after fractionating the mucus samples in <10 kDa and ≥10 kDa fractions. In the case of samples categorized as <10 kDa and ≥10 kDa, a volume of 10 μL was assayed for both. Antioxidant activity of the <10 kDa fraction was higher than that of the ≥10 kDa fraction (40.0 ± 8.3% versus 27.3 ± 4.6%; Figure 1C), although the results were not statistically different (*p* = 0.05).

### 2.3. Anti-Inflammatory Activity

The anti-inflammatory potential of the mucus and the <10 kDa fraction was evaluated using the COX (ovine/human) Inhibitor Screening Assay Kit (Item No. 560131, Cayman Chemical, Ann Harbor, Michigan, USA), and results were presented as COX inhibition percentages. Ten μL of mucus samples exhibited an inhibition value of 36.4 ± 16.8% for COX-1, but no measurable activity for COX-2. On the other hand, 10 μL of the mucus fractions with <10 kDa exhibited inhibition values of 37.9 ± 8.8% for COX-1 and 36.2 ± 16.5% for COX-2.

## 3. Discussion

### 3.1. Antimicrobial Activity

Mucus samples from the sea slug *E. crispata* showed inhibitory activity against several bacterial strains tested, with a higher inhibitory effect against Gram–negative bacteria. Particularly relevant was the high inhibitory activity against three strains of *Pseudomonas aeruginosa*. This Gram–negative bacteria is listed in the World Health Organization (WHO) Bacterial Priority Pathogens List (BPPL) of 2024 in the high-priority group [22]. Bacterial pathogens included in this high-priority group are responsible for difficult-to-treat infections, cause substantial disease burden (mortality and morbidity), show increasing trends in resistance, are difficult to control in clinical settings, and are highly transmissible. Only a few potential treatments for these pathogens are in the development pipeline, and they can be critical for some populations and in specific geographical areas [22].

As an opportunistic pathogen occurring widely in the environment, *P. aeruginosa* causes blood, lung, urinary tract, and kidney infections, being especially relevant for immunocompromised individuals and in health-care settings [23]. Risks posed by *P. aeruginosa* are related to its outer polysaccharide layer, multidrug efflux transporters, and high level of biofilm formation, together leading to a high probability of antibiotic resistance [24,25,26]. Marine natural products isolated from marine plants, animals, and microorganisms have the potential to be used as agents against *P. aeruginosa*. Over the last decade, approximately 80 natural compounds with potential applications against this pathogen were obtained from marine microbes, invertebrates, and fish, including polyketides, alkaloids, peptides, and terpenoids [23]. Similar antimicrobial activity was observed for native protein extracts, indicating that proteins present in the mucus of *E. crispata* contributed significantly to the observed activity.

Previously, we performed a comprehensive analysis of the protein composition of the mucus from *E. crispata* that revealed a diverse array of 306 proteins [21]. The full protein dataset is available at PRIDE Database at https://doi.org/10.6019/PXD042643 (submitted 2 June 2023). A significant proportion of these proteins exhibited enzymatic activities, notably hydrolases, which are well-known for their antimicrobial properties [27]. This suggests that the mucus of *E. crispata* might play a role in its defense mechanisms against bacterial pathogens, contributing to its bioactive properties [21]. Among the most abundant proteins present in the mucus, a potential candidate for the observed activity against *P. aeruginosa* is lysozyme g (A0A433TQC7). This protein is part of a group of hydrolytic enzymes characterized by its ability to cleave the β-(1,4)-glycosidic bonds in peptidoglycan, a major structural component of the bacterial cell wall [28,29]. Other potentially relevant proteins found in the mucus were peroxidases, which can generate reactive products with a wide range of antimicrobial activities [30]. In conclusion, it is possible that the antimicrobial activity observed for *E. crispata* mucus was caused by the interaction of several molecules present in the mucus samples.

Among the antimicrobial compounds of marine origin that have been previously described to display inhibitory activity against *P. aeruginosa* is myxinidin, a peptide discovered from acidic epidermal mucus extract of hagfish *Myxine glutinosa* [31]. Extracts of the mucus cocoon of parrotfish (*Scarus* sp.) were also shown to display antimicrobial activity against a variety of Gram–negative bacteria, including *P. aeruginosa* [32]. The mucus of terrestrial snail *Helix aspersa* was shown to exhibit inhibitory activity against *P. aeruginosa*, with size separation experiments indicating that the antimicrobial substances were between 30 and 100 kDa [33]. The mucus of the snails *Archachatina marginata* and *Achatina fulica* showed antimicrobial activity against *Staphylococcus*, *Streptococcus*, and *Pseudomonas* isolated from patients with wound infections, with an inhibitory effect comparable to that of seven different antibiotics used as control [34].

Polyketides are secondary metabolites with potential antimicrobial activity. Several polyketides of marine origin were shown to have broad antimicrobial activities against multiple clinical pathogens, including *P. aeruginosa* [35,36,37,38]. Polypropionate pyrones produced by sacoglossan sea slugs, including *E. crispata*, are among the most structurally complex isolated polyketides [39,40,41]. Further studies are required to assess the antimicrobial activity and biotechnological potential of these animal-derived polyketides.

Both the concentrated mucus and native protein extracts showed high inhibitory activities against multidrug resistant *P. aeruginosa* strain Z25.1, isolated from a diabetic foot infection. Diabetic foot infections are one of the most frequent complications that can arise in individuals suffering from diabetes, and, together with *S. aureus*, *P. aeruginosa* is one of the most common causes [42,43]. The main problems with *P. aeruginosa* diabetic foot infections include their association with a high proportion of multidrug resistant and biofilm producer strains, which increase the expression of inflammatory factors, eventually leading to vascular system damage, delayed wound healing and sepsis [43].

### 3.2. Antioxidant Activity

Reactive oxygen species such as free radicals may cause oxidative damage to macromolecules, such as DNA, proteins, and lipids [44]. This process plays a major part in the development of several chronic and degenerative diseases. Cells use antioxidants to counteract oxidative stress, reacting directly with free radicals or indirectly by inhibiting the activity/expression of free radical generating enzymes or enhancing the activity/expression of intracellular antioxidant enzymes [45]. Hence, antioxidants neutralize the excess of free radicals, protect the cells against their toxic effects, and contribute to disease prevention [44].

Marine organisms such as bacteria, microalgae, seaweeds, invertebrates and fish are rich sources of natural antioxidants, such as peptides, polysaccharides, terpenes, and polyphenolic compounds [46,47,48]. We observed inhibition of the ABTS radical by the mucus of the sea slug *E. crispata,* demonstrating antioxidant potential (40 μL of mucus 68.9 ± 7.7%, Figure 1A). Furthermore, the antioxidant activity was associated with the lower molecular weight fraction (10 μL <10 kDa; 40.0 ± 8.3% Figure 1C) of the mucus rather than to the native protein extracts. Low molecular weight compounds in terrestrial snail mucus, such as vitamins C and E, phenolic compounds, uric acid, and uronic acid, have antioxidant properties [49]. For example, relevant total antioxidant activity, assessed with the phosphomolybdate assay, was reported for the mucus of the land snail *Lissachatina fulica* and the freshwater snail *Pomacea canaliculata* [50]. Mucus-based gold nanoparticles obtained from the snail *H. aspersa* were found to be a potential ingredient in cosmetics, particularly due to its antioxidant activity [51].

### 3.3. Anti-Inflammatory Activity

The mucus of *E. crispata* exhibited pro-inflammatory COX-1 inhibition capacity (36.4 ± 16.8%), while the <10 kDa fraction showed inhibitory activity of both COX isoforms (37.9 ± 8.8% for COX-1 and 36.5 ± 16.5% for COX-2), indicating the presence of anti-inflammatory constituents. Several compounds with anti-inflammatory properties have been isolated from marine organisms such as bacteria, fungi, sponges, algae, and corals [52]. Compounds such as dysiarenone and deacetylphylloketal, extracted from marine sponges, were shown to have high anti-inflammatory activity, with inhibition of COX-2 expression [53,54]. Similarly, fucose-containing sulfated polysaccharides extracted from brown macroalgae displayed high anti-inflammatory potential, with inhibitory activity against both COX-1 and COX-2 [55,56]. Additionally, a diversity of compounds extracted from corals have shown promising results as anti-inflammatory metabolites [57,58,59]. Few studies have assessed the anti-inflammatory activity of mucus obtained from molluscs. Nonetheless, mucus obtained from the terrestrial snail *H. aspersa* demonstrated the ability to decrease the expression of pro-inflammatory COX-2, indicating its potential as an anti-inflammatory agent [60]. Comparing the mucus extracts of *H. aspersa* and the desert snail *Eremina desertorum*, El-Zawawy and Mona [61] showed that the mucus of the latter species was more effective as an antimicrobial and anti-inflammatory agent in the treatment of burn wound infections.

### 3.4. Concluding Remarks

This study represents the initial step to evaluate the potential biotechnological applications of the mucus derived from the tropical sea slug *E. crispata.* Its antimicrobial activity against *P. aeruginosa* is particularly noteworthy, paving the way for further investigations to pinpoint the specific compounds responsible for this inhibitory effect. Moreover, we also observed antioxidant and anti-inflammatory properties in the sea slug mucus. The reported capacity for gastropod mucus to facilitate wound healing [62] might be related to the combination of these multiple effects (antimicrobial, antioxidant and anti-inflammatory), and might be relevant in a scenario of increasing microbial resistance to antibiotic treatments. Understanding the bioactivities of gastropod mucus is an active and growing area of research and additional work is imperative to fully grasp and capitalize on the biotechnological applications of the molecules present in the mucus of *E. crispata*.

## 4. Materials and Methods

### 4.1. Animal Rearing

Adult *Elysia crispata* specimens were reared under laboratory conditions as previously described by Cartaxana et al. [63]. Animals were maintained in 150 L recirculated life-support systems (LSS), filled with artificial seawater (ASW), and kept at a salinity of 35 ppt and a temperature of 25 °C. The ASW was prepared by mixing salt (Red Sea Europe, Verneuil d’Avre et d’Iton, France) with reverse osmosis water (TMC, V2 Pure Advanced Reverse Osmosis System) following manufacturer instructions to achieve the desired salinity. The LSS was equipped with T5 fluorescent lamps, set to a photon scalar irradiance of 60 µmol photons m^−2^ s^−1^ measured at the water surface with a Spherical Micro Quantum Sensor and a ULM-500 Universal Light Meter (Heinz Walz GmbH, Effeltrich, Germany), running on a photoperiod of 12 h light:12 h dark.

The sea slugs were fed with *Bryopsis plumosa* (strain KU-0990) obtained from the Kobe University Macro-Algal Culture Collection in Japan. The algae were cultivated at 20 °C in 2 L flasks containing ASW and f/2 medium, devoid of silica, and provided with constant aeration. Algae were cultured at an irradiance of 60–80 μmol photons m^−2^ s^−1^ provided by LED lamps (Valoya 35 W, spectrum NS12, Helsinki, Finland), with a 12 h light:12 h dark photoperiod.

### 4.2. Mucus Collection

Mucus was collected as previously described by Lopes et al. [21]. Briefly, sea slugs were collected from the LSS and rinsed with clean ASW to remove any debris attached to their bodies. Subsequently, each slug was carefully placed in a 200 μm sieve and excess water was removed with tissue paper underneath the sieve. The slugs were then placed in individual 8 cm watch glasses, wetted with 200 µL of ASW, and gently swirled for 5 min to induce mucus production. The secreted mucus was collected with a micropipette into previously cooled Eppendorf tubes. Individual samples, approximately 1.5–2.0 mL, corresponded to the mucus secreted by a pool of 7 animals.

Bioactivities were assessed in concentrated mucus samples. For this, mucus samples were subjected to a 2 h partial drying process using a speed vacuum (Savant SPD121P SpeedVac Concentrator, Thermo Fisher Scientific, Bremen, Germany). The duration of operation was selected to achieve approximately half the original sample volume. Samples were then frozen at −80 °C until further processing.

### 4.3. Native Protein Extract

Native protein extracts were obtained following the protocol described by Elder et al. [64], with some modifications. Extraction buffer (1 M Tris-HCl buffer, pH 7.5) was added to the concentrated mucus samples to achieve a final concentration of Tris-HCL buffer of 50 mM. Samples were centrifuged at 10,000× *g* for 30 min at 4 °C to remove debris. The supernatant was collected, and the soluble proteins were precipitated by slowly adding solid ammonium sulfate to achieve 70% saturation. The samples were gently stirred overnight at 4 °C and centrifuged at 25,000× *g* for 20 min at 4 °C. The resulting pellets were suspended in extraction buffer (50 mM Tris-HCl buffer, pH 7.5) and quantified using the RC/DC protein assay kit (Bio-Rad, Hercules, CA, USA) following the manufacturer’s protocol.

### 4.4. Fractions with <10 kDa and ≥10 kDa

Concentrated mucus samples were divided into two fractions: (i) compounds with molecular weight lower than 10 kDa, and (ii) compounds with molecular weight equal to or higher than 10 kDa. Samples were transferred to Amicon Ultra-0.5 Centrifugal Filter Units (10 kDa, Millipore, Burlington, MA, USA), following the manufacturer’s recommendations. Each device is supplied with two microcentrifuge tubes. During the procedure, one tube was used to collect the filtrate, and the other to recover the pellet. The sample (500 µL) was added to the Amicon Ultra-0.5 mL device and then centrifuged at 14,000× *g* for 30 min until no volume was left. Samples were kept at −80 °C until further analysis.

### 4.5. Antimicrobial Activity

The bacterial inhibitory capacity of concentrated mucus samples was evaluated against a collection of 17 pathogenic bacterial strains from the Laboratory of Microbiology and Immunology of the Faculty of Veterinary Medicine of the University of Lisbon, as detailed in Table 3. The antibacterial activity was determined through a spot-on-lawn test method [65].

First, the bacteria were inoculated on Brain Heart Infusion (BHI) agar plates and incubated at 37 °C for 24 h. Subsequently, microbial suspensions with a concentration of 1 × 10^8^ CFU mL^−1^ were prepared in sodium chloride and inoculated on the surface of Mueller–Hinton agar plates to form a uniform lawn of microbial growth. Next, 10 µL of mucus samples were spotted on the surface of each microbial lawn and incubated for 24 h at 37 °C, with 4 biological replicates being tested. Following incubation, the plates were observed for the development of inhibition halos at the spot where the samples were placed.

**Figure 2:**
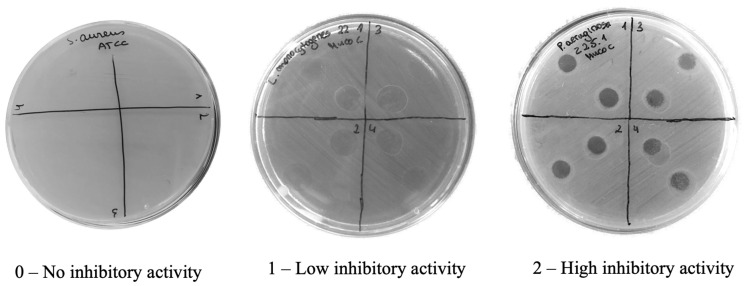
Illustrative examples of inhibition across three levels of the initial assessment scale: 0—no inhibitory activity; 1—low inhibitory activity, and 2—high inhibitory activity. Ten µL droplets of concentrated mucus or mucus native protein extracts were added to the bacterial mats.

The inhibitory effect was initially graded on a scale from 0 (no inhibitory effect), 1 (low inhibitory effect) to 2 (high inhibitory effect) (Figure 2). The average inhibition levels from multiple replicates were calculated, leading to the creation of a refined scale: (–) for 0 ≤ to < 0.5 (no inhibitory effect), (+) for 0.5 ≤ to < 1 (low inhibitory effect), (++) for 1 ≤ to < 1.5 (medium inhibitory effect), and (+++) for ≥1.5 (high inhibitory effect).

Subsequently, seven strains were chosen to evaluate the native protein-mediated inhibition of bacterial growth (Table 3). Similarly, to the mucus samples, 10 µL of resuspended protein extracts were spotted on the surface of each microbial lawn and incubated for 24 h at 37 °C, with 4 biological replicates being tested. Incubation and evaluation of the inhibitory effects were performed as described above.

All tests were performed in duplicate, and two independent assays were performed in different days.

### 4.6. Antioxidant Activity

The antioxidant scavenging activity against the 2,2′-azino-bis-3-ethylbenzothiazoline-6-sulfonic acid radical cation (ABTS^●+^) was evaluated using the method described by Re et al. [66], with some modifications. Trolox was used as a standard solution, prepared at concentrations of 5, 12.5, 25, and 37.5 µM in ethanol.

Antioxidant scavenging activity was tested in concentrated mucus, native protein extracts and <10 kDa and ≥10 kDa fractions. In all cases, three biological replicates were tested. The assay volumes tested for concentrated mucus samples were: 5, 10, 20 and 40 µL, while for native proteins and <10 kDa and ≥10 kDa fractions, a total amount of 10 µL was used. Protein samples in native conditions were tested using protein concentrations of 1, 2, and 4 μg μL^−1^. Samples and standards were mixed with 190 µL of ABTS^●+^ working solution in ammonium persulfate (APS) (with an initial absorbance of approximately 0.9 at 734 nm) in triplicate. The mixture was then incubated for 120 min, and the absorbance was measured at 734 nm every 5 min using a Multiskan GO 1.00.38 spectrophotometer (Thermo Scientific, Hudson, NH, USA).

A sample blank was prepared for each sample by replacing the ABTS^●+^ solution with 5 mM PBS (phosphate-buffered saline) solution. The antioxidant activity was calculated in terms of the percentage of inhibition of the ABTS radical using the following Equation (1):

(1) $$ABTS\;Inhibition\left(\%\right)=\left(\frac{ControlAbs-SamplesAbs}{ControlAbs}\right)\times 100$$

### 4.7. Anti-Inflammatory Activity

The anti-inflammatory activity was assessed using the COX (ovine/human) Inhibitor Screening Assay Kit, following the manufacturer’s recommendations. To prepare the assay, 20 μL of COX-1 (ovine) and COX-2 (human recombinant) were inactivated in boiling water for 3 min to generate the background values of each enzyme. Then, 160 μL of reaction buffer provided in the kit, 10 μL of heme, and 10 μL of the previously inactivated COX-1 or COX-2 were added to Eppendorf tubes labelled as background COX-1 (BC1) and background COX-2 (BC2). Tubes for 100% initial activity (IA) for both COX-1 and COX-2 were prepared by adding 160 μL of reaction buffer, 10 μL of heme, and 10 μL of active COX-1 or COX-2.

Inhibitor tubes were prepared for concentrated mucus and <10 kDa fractions. For concentrated mucus samples, only COX-1 was assayed as preliminary trials showed no inhibitory activity for COX-2 within the recommended cut-off values by the manufacturer. In each inhibitor sample tube, 160 μL reaction buffer, 10 μL of heme, 10 μL of COX-1 or COX-2 enzyme, and 10 μL of the inhibitor solution were added. All solutions were incubated for 10 min at 37 °C. After incubation, 10 μL of arachidonic acid was added to all test tubes and briefly mixed, followed by incubation for 2 min. Then, 50 μL of 1 M hydrochloric acid was added to stop the reaction, followed by the addition of 100 μL of stannous chloride solution to each test tube. Samples were mixed and incubated for 15 min at 4 °C. To accommodate the assay’s usable range, dilutions for the background samples, 100% initial activity samples, and inhibitor samples were performed as follows: 1:100 for background samples; 1:2000 and 1:4000 for both 100% initial activity and inhibitor samples.

Prostaglandin screening standards were prepared at eight concentrations (S1–S8). A volume of 900 μL of Elisa buffer was added for S1 and 500 μL for standards S2 to S8. Then, 100 μL of bulk standard (10 ng mL^−1^) was added to tube S1 and mixed thoroughly. The standards were serially diluted by transferring 500 μL from tube S1 to tube S2 and mixed thoroughly. This process was repeated until S8. Samples were added to a 96-well plate provided by the manufacturer and incubated at 4 °C for 18 h. After incubation, the plate was developed by emptying the wells and rinsing them with wash buffer five times. Then, 200 μL of Ellman’s reagent was added to each well, and the plate was covered with plastic film and kept in an orbital shaker for 90 min. The absorbances were read at 90, 100, 110, and 120 min at several wavelengths: 405, 410, 415, and 420 nm using a Synergy™ HTX Multi-Mode Microplate reader (BioTek^®^, Winooski, VT, USA).

Three biological replicates were tested for concentrated mucus and <10 kDa fractions.

### 4.8. Statistical Analysis

The existence of a significant effect of mucus volume on the antioxidant activity was tested using one-way ANOVA. Normality was checked using Shapiro–Wilk test, and homogeneity of variances using Levene’s test. Multiple comparisons were performed using Tukey’s test. A Kruskal–Wallis non-parametric test was performed to test the effect of mucus native protein concentration on the antioxidant activity, as data failed to meet ANOVA assumptions. A significant difference between the antioxidant activity of mucus size fractions <10 kDa and ≥10 kDa was tested using Mann–Whitney U test. Statistical analyses were carried out using IBM SPSS Statistics 24.

## Figures and Tables

**Table 1:** Assessment of the antimicrobial activity of the mucus of the sea slug *Elysia crispata* against 17 bacterial isolates. The following inhibition scale was used: (–) no inhibitory effect; (+) low inhibitory effect; (++) medium inhibitory effect; and (+++) high inhibitory effect, as detailed in the Materials and Methods section.

Bacterial Isolates		Inhibitory Effect
*Enterococcus faecalis* ATCC 51299	Gram +	+
*Enterococcus faecium* CCUG 36804		+
*Staphylococcus aureus* ATCC 29213		–
*Staphylococcus aureus* Z25.2		+
*Staphylococcus pseudintermedius* 93/23		–
*Streptococcus equi*		+
*Bacillus anthracis*		+
*Listeria monocytogenes* 22		++
*Listeria monocytogenes* CECT 935		++
*Escherichia coli* ATCC 25922	Gram –	+
*Pseudomonas aeruginosa* ATCC 27853		+++
*Pseudomonas aeruginosa* Z25.1		+++
*Pseudomonas aeruginosa* 74/23		+++
*Aeromonas hydrophila* ATCC 7966		++
*Salmonella enterica* CECT 443		+
*Salmonella* Rissen		+
*Proteus mirabilis* 250/23		+

**Table 2:** Assessment of the antimicrobial activity of the native protein extracts obtained from the mucus of the sea slug *Elysia crispata* against seven bacterial isolates. The following inhibition scale was used: (–) no inhibitory effect; (+) low inhibitory effect; (++) medium inhibitory effect; and (+++) high inhibitory effect, as detailed in the Materials and Methods section.

Bacterial Isolates		Inhibitory Effect
*Staphylococcus aureus* ATCC 29213	Gram +	+
*Staphylococcus aureus* Z25.2		+
*Listeria monocytogenes* 22		++
*Listeria monocytogenes* CECT 935		+
*Pseudomonas aeruginosa* ATCC 27853	Gram –	+++
*Pseudomonas aeruginosa* Z25.1		+++
*Pseudomonas aeruginosa* 74/23		+++

**Table 3:** Bacterial isolates and respective origin used to test the inhibitory capacity of mucus and native protein extracts of the mucus of *Elysia crispata*. * indicates the bacterial isolates used to test the antimicrobial activity of native protein extracts.

Bacterial Isolates	Origin
*Escherichia coli* ATCC 25922	Culture collection
*Enterococcus faecalis* ATCC 51299	Culture collection
*Enterococcus faecium* CCUG 36804	Culture collection
*Staphylococcus aureus* ATCC 29213 *	Culture collection
*Staphylococcus aureus* Z25.2 *	Diabetic Foot Infection
*Staphylococcus pseudintermedius* 93/23	Canine Urinary tract infection
*Pseudomonas aeruginosa* ATCC 27853 *	Culture collection
*Pseudomonas aeruginosa* Z25.1 *	Diabetic Foot Infection
*Pseudomonas aeruginosa* 74/23 *	Canine otitis externa
*Aeromonas hydrophila* ATCC 7966	Culture collection
*Salmonella enterica* CECT 443	Culture collection
*Salmonella* Rissen	Swine
*Streptococcus equi*	Culture collection
*Bacillus anthracis*	Culture collection
*Proteus mirabilis* 250/23	Culture collection
*Listeria monocytogenes* 22 *	Culture collection
*Listeria monocytogenes* CECT 935 *	Culture collection

## Data Availability

The original contributions presented in the study are included in the article/Supplementary Materials, further inquiries can be directed to the corresponding author.

## Supplementary Materials

The following supporting information can be downloaded at: https://www.mdpi.com/article/10.3390/molecules29194593/s1, Raw_Data_Sheet.
